# Expression analysis of the osteoarthritis genetic susceptibility mapping to the matrix Gla protein gene *MGP*

**DOI:** 10.1186/s13075-019-1934-7

**Published:** 2019-06-18

**Authors:** Colin Shepherd, Abigail E. Reese, Louise N. Reynard, John Loughlin

**Affiliations:** Skeletal Research Group, Institute of Genetic Medicine, Newcastle University, International Centre for Life, Newcastle upon Tyne, NE1 3BZ UK

**Keywords:** Osteoarthritis, Genetic association signal, *MGP*, Allelic expression, RNA interference, Knockdown

## Abstract

**Background:**

Osteoarthritis (OA) is a common disease of older individuals that impacts detrimentally on the quality and the length of life. It is characterised by the painful loss of articular cartilage and is polygenic and multifactorial. Genome-wide association scans have highlighted over 90 osteoarthritis genetic signals, some of which reside within or close to highly plausible candidate genes. An example is an association to polymorphisms within and adjacent to the matrix Gla protein gene *MGP*. We set out to undertake a functional study of this gene.

**Methods:**

Nucleic acid was extracted from cartilage, infrapatellar fat pad, synovium, trabecular bone, trapezium and peripheral whole blood from OA patients and also from mesenchymal stem cells (MSCs) subjected to chondrogenesis. Expression of *MGP* was measured by quantitative PCR (qPCR), RNA-sequencing and allelic expression imbalance (AEI) analysis. Matrix Gla protein was depleted in chondrocytes by knocking down *MGP* expression using RNA interference (RNAi) and the effect on a range of genes assessed by qPCR.

**Results:**

*MGP* is expressed in joint tissues, blood and chondrocytes cultured from MSCs. There is a higher expression in diseased versus non-diseased cartilage. Polymorphisms that are associated with OA also correlate with the expression of *MGP*, with the OA risk-conferring allele showing significantly reduced expression in cartilage, fat pad and synovium but increased expression in blood. Depletion of Matrix Gla protein had a significant effect on the majority of genes tested, with an increased expression of catabolic genes that encode enzymes that degrade cartilage.

**Conclusions:**

*MGP* expression is subject to *cis*-acting regulators that correlate with the OA association signal. These are active in a range of joint tissues but have effects which are particularly strong in cartilage. An opposite effect is observed in blood, highlighting the context-specific nature of the regulation of this gene’s expression. Recapitulation of the genetic deficit in cartilage chondrocytes is pro-catabolic.

**Electronic supplementary material:**

The online version of this article (10.1186/s13075-019-1934-7) contains supplementary material, which is available to authorized users.

## Introduction

Osteoarthritis (OA) is a highly polygenic musculoskeletal disorder that is common in most populations and principally affects older individuals. It is characterised by a gradual, focal loss of the articular cartilage with concurrent and consequential alterations to other joint tissues [1]. Patients initially present with chronic joint pain and loss of normal joint function with surgical intervention often required in severe cases of the disease. OA therefore impacts on the quality of life, but it can also shorten the length of life [2].

Genome-wide association scans (GWAS) have reported over 90 independent genome-wide significant risk alleles for OA [3–7]. Most of these studies have focused on hand, knee or hip OA, with disease at these sites having a particularly large impact on the health of the patient and on health care systems.

The principal translational goal of OA GWAS studies is the generation of new knowledge regarding disease aetiology that can then be directed to treatment development. In this regard, the association with hand OA of functional polymorphism at the *MGP* locus was very noteworthy [8]. This gene codes for matrix Gla protein, which is secreted by cartilage chondrocytes into the synovial joint space, where it regulates the levels of free calcium via the affinity of its γ-carboxyglutamic acid (Gla) residues for calcium ions [9]. In so doing, matrix Gla protein inhibits ectopic calcification of the joint. The OA association was to single nucleotide polymorphism (SNP) rs4764133 (C > T), which is located upstream of *MGP* and which correlated with altered expression of the gene in cartilage; the OA-risk conferring T allele of rs4764133 showed reduced expression relative to the non-risk C allele [8]. This implies that the genetic susceptibility at this locus acts by lowering the level of *MGP* expression, which leads to reduced amounts of matrix Gla protein. This would be permissive to ectopic cartilage calcification and as such may be the mechanism by which this association signal acts to increase OA risk. This genetic data supports the application in OA of compounds that could attenuate this calcification, such as vitamin K which is involved in the biosynthesis of Gla-rich proteins [10].

The compelling and potentially translatable nature of the *MGP* genetic association report prompted us to undertake a more detailed molecular analysis of this signal. We set out to replicate and then expand on the *MGP* expression study into other joint tissues and cells from patients. We also modelled the impact of the rs4764133 risk-conferring allele by knocking down *MGP* in cartilage chondrocytes and assessing effects on anabolic, catabolic, and hypertrophic genes.

## Materials and methods

### OA patients

Joint tissue samples were obtained from patients undergoing orthopaedic surgical procedures at the Newcastle upon Tyne NHS Foundation Trust hospitals. Samples were collected from two categories of patient: (1) those with primary hip or knee OA who had undergone joint replacement surgery and (2) those with primary hand OA who had undergone a trapeziectomy. In total, we accessed and studied tissue samples from a total of OA 165 patients.

For knee and hip OA patients, we collected macroscopically normal cartilage (distal from the lesion and therefore avoiding areas of fibrillated tissue), infrapatellar fat pad, synovium and trabecular bone (which were taken from non-sclerotic areas). For some of our knee and hip patients, more than one joint tissue type was available for analysis. For hand OA patients, cartilage could not be separated from subchondral bone due to the small size of the trapeziectomy samples, and as such, subchondral bone with its attached cartilage (osteochondral samples) was collected. We also collected whole peripheral blood samples from some of our hip and knee OA patients just prior to their surgery, using EDTA vacutainers for DNA extraction and Tempus™ tubes for RNA extraction (ThermoFisher Scientific). Further details regarding the patients can be found in Additional file 1: Table S1.

### Nucleic acid extractions

Tissue samples were stored at − 80 °C and ground to a powder using a mixermill (Retsch Limited) under liquid nitrogen. For cartilage, bone and trapeziectomy samples, RNA was extracted using TRIzol (Life Technologies) and DNA was extracted using the E.Z.N.A. Tissue DNA isolation kit (Omega Biotek, VWR). For synovium and fat pad, DNA and RNA were extracted using the E.Z.N.A. DNA/RNA isolation kit (Omega Biotek, VWR). For blood, DNA was extracted using the QIAamp DNA blood mini kit (Qiagen) and RNA extracted using the Tempus™ Spin RNA isolation kit (ThermoFisher Scientific).

### Quantitative gene expression

cDNA synthesis and quantitative PCR (qPCR) were performed as described previously [11]. Predesigned TaqMan assays (Integrated DNA Technologies) or assays designed using the Roche probe library system were used to quantify expression of the housekeeping genes *HPRT1*, *18S* and *GAPDH* and 12 target genes. The relative gene expression was calculated by the 2^−ΔCt^ method, where ΔCt is the mean Ct value of the three housekeeping genes subtracted from the Ct value of the target gene of interest. For target gene *RUNX2*, two qPCR assays were used: one targeting exons 6–7, measuring both the main isoforms of the gene (termed *RUNX2all*), and one targeting exons 1–2, which measures the longer isoform of the gene (termed *RUNX2long*) [12].

### Mesenchymal stem cell (MSC) chondrogenesis

Immortalised human MSCs derived from adipose tissue (SCRC-4000, ATCC) were subjected to V-bottom 96-well-plate pellet chondrogenesis for 21 days, as described previously [13]. Human bone marrow MSCs from a female donor aged 24 years (Lonza Biosciences) were cultured, phenotype tested and subjected to V-bottom 96-well-plate pellet chondrogenesis for 7 days, as described previously [14]. In each case, three pellets were analysed at each time point, representing three independent differentiations. RNA was extracted from each pellet using TRIzol (Life Technologies), cDNA synthesised and *MGP* expression measured by qPCR with three technical repeats per cDNA.

### RNA-sequencing (RNA-seq)

The expression of *MGP* was assessed using RNA-seq data that we had previously generated from the cartilage of ten hip OA patients and six patients who had undergone hip replacement due to a neck-of-femur (NOF) intracapsular fracture [15]. The NOF patients showed no signs or symptoms of hip OA, and their cartilage was macroscopically intact and with no lesions. Transcripts per million (TPM) values were extracted using R (http://www.R-project.org/) and visualised using the ggplot2 library in R. Differential expression analysis between OA and NOF was carried out with the Bioconductor package DESeq2 [16].

### Genotyping

We chose to analyse *MGP* transcript SNP rs4236 (T > C, OA risk allele = C, minor allele frequency = 0.37) as the difference in expression between its risk and non-risk alleles was the largest of those SNPs investigated in the original study [8]. The SNP was genotyped by pyrosequencing using the primers listed in Additional file 2: Table S2. The assay was designed using PyroMark assay design 2.0 (Qiagen), and the sequencing was performed using the PyroMark Q24 Advanced platform (Qiagen).

### Allelic expression imbalance (AEI)

AEI at rs4236 heterozygotes was quantified by pyrosequencing, using the methodology described above and the same primers. The sequences were generated automatically and an output of allelic ratio was produced using PyroMark Advanced software (Qiagen). For each cDNA and DNA sample from a heterozygote, PCR reactions were formed in triplicate. Samples were excluded from the analysis if the values between the PCR replicates differed by > 5%. The respective cDNA and DNA were analysed concurrently and allelic expression of cDNA was normalised to its corresponding DNA.

### RNA interference (RNAi)

Primary human articular chondrocytes (HACs) were isolated by enzymatic digestion of OA knee cartilage, cultured, and RNAi was then performed, essentially as described previously [11, 17]. The cells were seeded in 6-well plates at a density of 350,000 cells per well. After 24 h, cells were transfected with 50 nM Dharmacon ON-TARGETplus SMARTpool small interfering RNA (siRNA) targeted against *MGP* (L-009770-00) or a non-targeting scrambled siRNA control (D-001810-10-20). Cells were harvested 48 h after transfection, and RNA and protein were extracted using the Nucleospin RNA/protein kit (Macherey-Nagel, supplied by ThermoFisher). Gene expression was assessed by qPCR using cDNA synthesised from RNA extracted from each well, with a minimum of four technical repeats per gene analysed. For immunoblot analysis of matrix Gla protein depletion, 10 μg of total protein was resolved on 10% (w/v) SDS–polyacrylamide gels. Blots were probed with anti-matrix Gla protein (α-MGP; Proteintech 10734-1-AP) or anti-GAPDH (α-GAPDH; Cell Signalling Technologies) antibodies. Matrix Gla protein depletion was quantified using ImageJ software [18] by normalising to levels of α-GAPDH.

### Statistical analyses

For quantitative gene expression analyses, *P* values were calculated using a Mann-Whitney 2-tailed exact test. For RNA-seq, hypothesis testing was performed using the DESeq2 implementation of the Wald test. For AEI, *P* values were calculated using a Mann-Whitney 2-tailed exact test. For gene expression analyses in the RNAi experiment, *P* values were calculated using a Student’s 2-tailed *t* test. For quantification of MGP protein abundance, *P* values were calculated using a Student’s 2-tailed *t* test.

## Results

### Expression of *MGP*

**Fig. 1:**
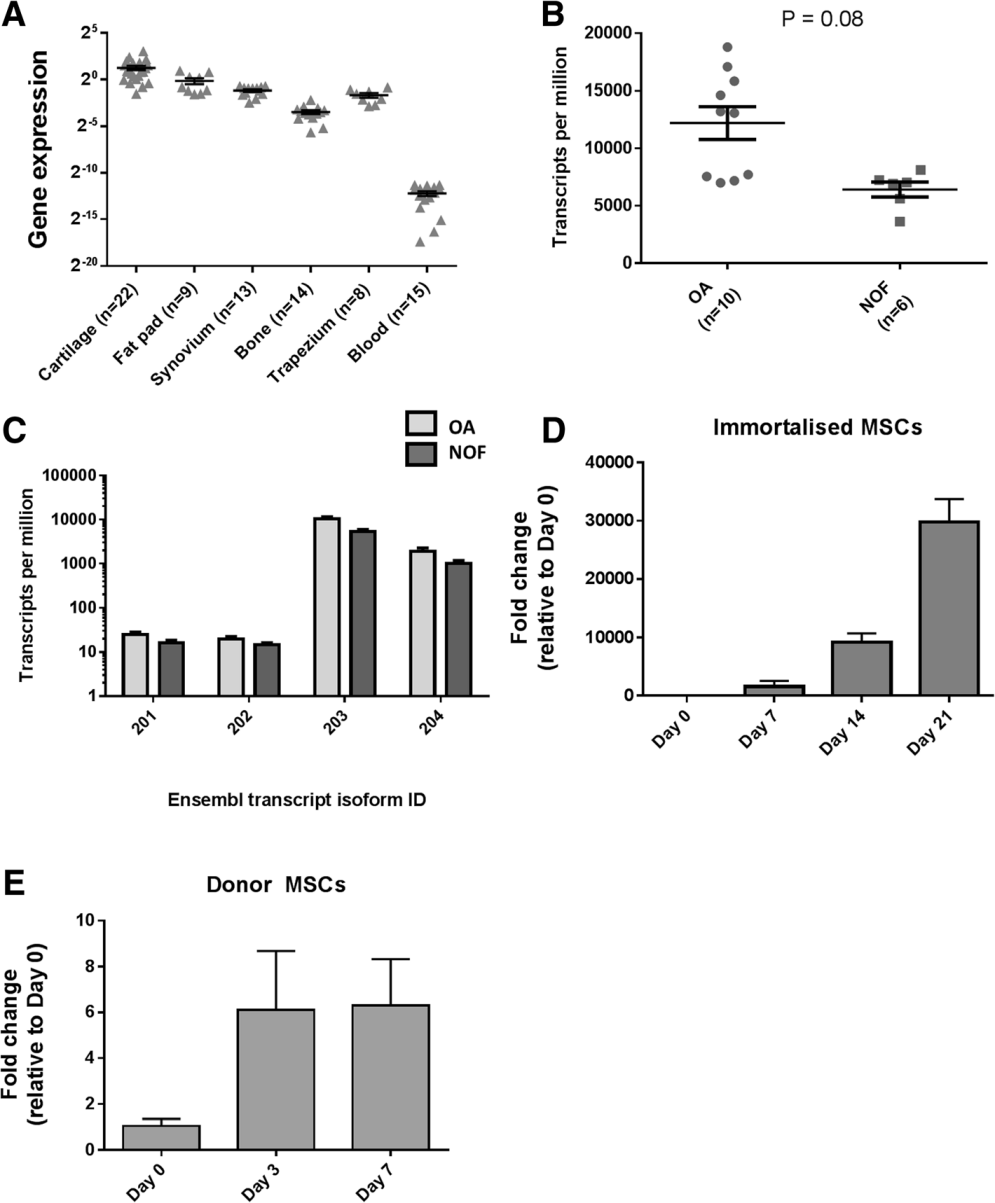
Expression analysis of *MGP* in multiple tissue samples from patients and during chondrogenesis. **a** Expression of *MGP* was measured by qPCR in OA patient cartilage, infrapatellar fat pad, synovium, trabecular bone, trapezium and blood. **b** Expression of *MGP* was measured in hip cartilage from OA patients and patients with a neck-of-femur (NOF) fracture as controls, using RNA-sequencing data. **c** Expression of *MGP* transcript isoforms (designated by their Ensembl database identification numbers) was measured using the same RNA-sequencing data. **d** Expression of *MGP* during chondrogenesis of immortalised human MSCs. **e** Expression of *MGP* during chondrogenesis of bone marrow MSCs from a human donor. In all panels, horizontal lines with bars show the mean ± standard error of the mean (SEM)

Using qPCR, we initially assessed the expression of *MGP* in five joint tissues from our OA patients: cartilage (*n* = 22), fat pad (*n* = 9), synovium (*n* = 13), trabecular bone (*n* = 14), and trapezium (*n* = 8). We also examined expression in peripheral blood (*n* = 15 patients). *MGP* was expressed in all samples, with the highest level being in cartilage and the lowest in blood (Fig. 1a). We next compared the level of *MGP* expression between hip cartilage from OA (*n* = 10) and NOF (*n* = 6) patients in our RNA-seq data. The gene was expressed in both patient groups with a non-significant (*P* = 0.08) higher level in OA (Fig. 1b). Using this RNA-seq data, we then assessed the relative expression levels of the transcript isoforms of *MGP*. The gene has four isoforms, three of which are protein coding (isoforms 201, 203 and 204) and one which is not (isoform 202). All were expressed in cartilage, with isoform 203, which codes for the canonical form of the protein, being most abundant (Fig. 1c). We next assessed the expression of *MGP* during pellet chondrogenesis, which was performed on an immortalised human MSC cell line (Fig. 1d) and on primary MSCs from a human donor (Fig. 1f). The gene showed an induction in both cell types, with a particularly striking increase in expression during chondrogenesis in the cell line.

### Allelic expression imbalance

**Fig. 2:**
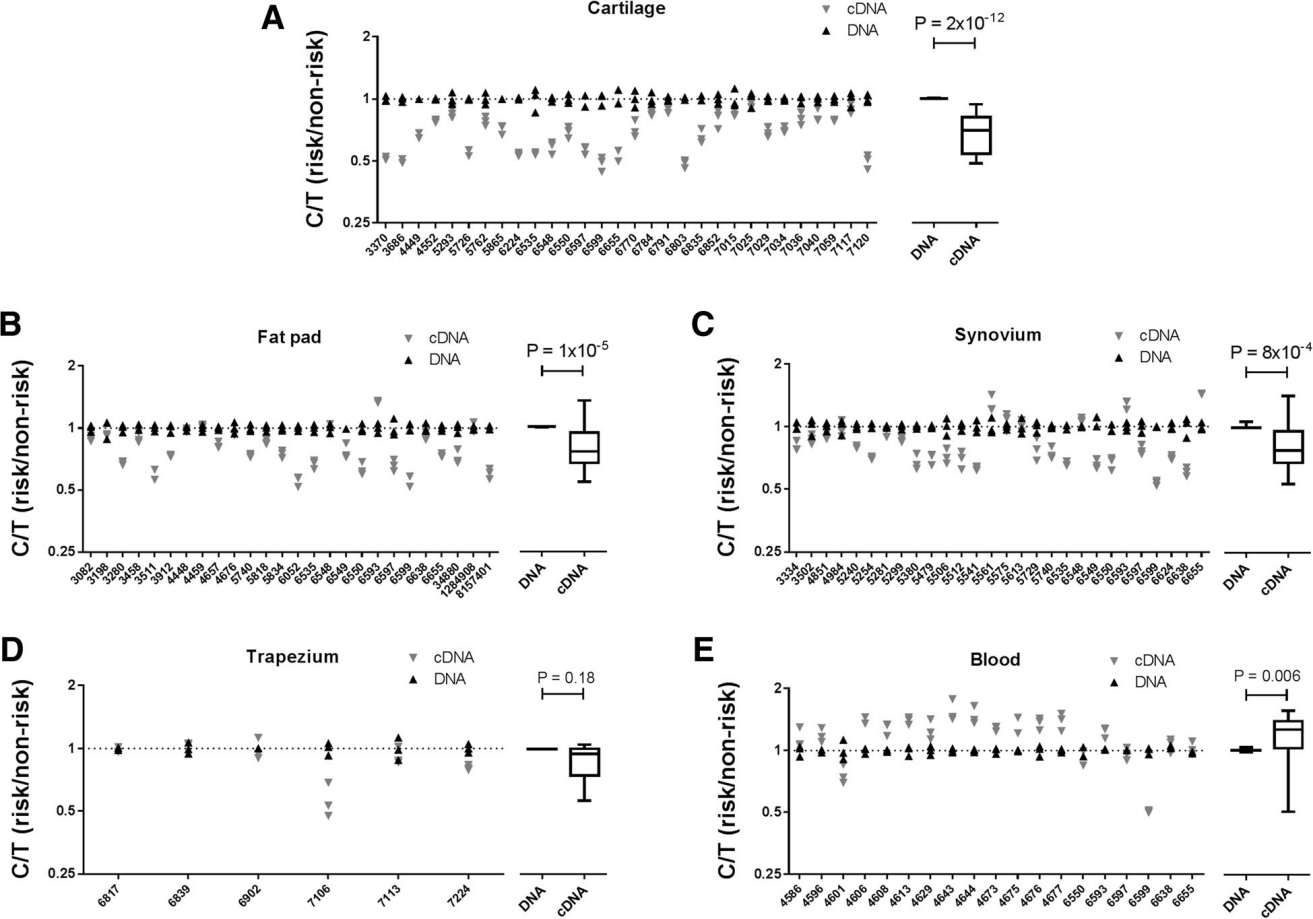
Allelic expression imbalance (AEI) analysis of *MGP*. AEI analysis of rs4236 was carried out in OA patient cartilage (*n* = 30) (**a**), infrapatellar fat pad (*n* = 26) (**b**), synovium (*n* = 28) (**c**), trapezium (*n* = 6) (**d**) and blood (*n* = 19) (**e**). The *y*-axis for each plot shows the risk/non-risk (C/T) allelic ratios, with a ratio < 1 indicating decreased expression and a ratio > 1 indicating increased expression of the C allele. Three technical repeats were performed for each patient’s DNA and cDNA. The right panels show the mean values for DNA and cDNA from all patients combined, with results represented by box-and-whisker plots, in which the lines within the box represent the median, the box represents the 25th to 75th percentiles, and the whiskers represent the minimum and maximum values. *P* values were calculated using a Mann-Whitney 2-tailed exact test. Individual patients are designated by their anonymised identification (ID) numbers

In the GWAS report for *MGP*, the authors used allelic expression imbalance (AEI) analysis to demonstrate that the OA risk conferring T allele of rs4764133 correlated with reduced expression of the gene in cartilage [8]. To do this, they studied three *MGP* transcript SNPs (rs4236, rs1049897 and rs1800801) that are in high linkage disequilibrium (LD) with each other and with rs4764133 (all pair wise *r*^2^ values > 0.85). Each transcript SNP had shown significant evidence of AEI. We chose to use rs4236 (T > C; *r*^2^ = 0.89 relative to rs4764133; T allele of rs4764133 is equivalent to the C allele of rs4236), the SNP that showed the largest degree of AEI in that initial report (39.6% of the OA risk-conferring C allele versus 60.4% of the non-risk T allele). This SNP is located within the final exon of *MGP* and is present in all four transcript isoforms. We genotyped our patients for rs4236 to identify heterozygotes. We then investigated AEI in cartilage (*n* = 30 patients), fat pad (*n* = 26 patients), synovium (*n* = 28 patients), trapezium (*n* = 6 patients) and blood (*n* = 19 patients) (Fig. 2). In cartilage, we observed AEI that was comparable to that seen in the original report; a mean AEI ratio (C/T) of 0.69, equivalent to an average of 40.8% of the C allele in the 30 patients combined (*P* = 2 × 10^−12^). In fat pad and synovium, we also observed a significant AEI, although not as striking as that seen in cartilage: a mean AEI ratio of 0.81, equivalent to 44.8% of the C allele, for fat pad (*P* = 1 × 10^−5^), and a mean AEI ratio of 0.84, equivalent to 45.7% of the C allele, for synovium (*P* = 8 × 10^−4^). In trapezium, there was a non-significant (*P* = 0.18) mean AEI ratio of 0.89, equivalent to 47.1% of the C allele, whilst in blood, the mean AEI ratio was in the opposite direction to that seen in the joint tissues: 1.19, which is equivalent to 54.3% of the C allele (*P* = 0.006).

**Fig. 3:**
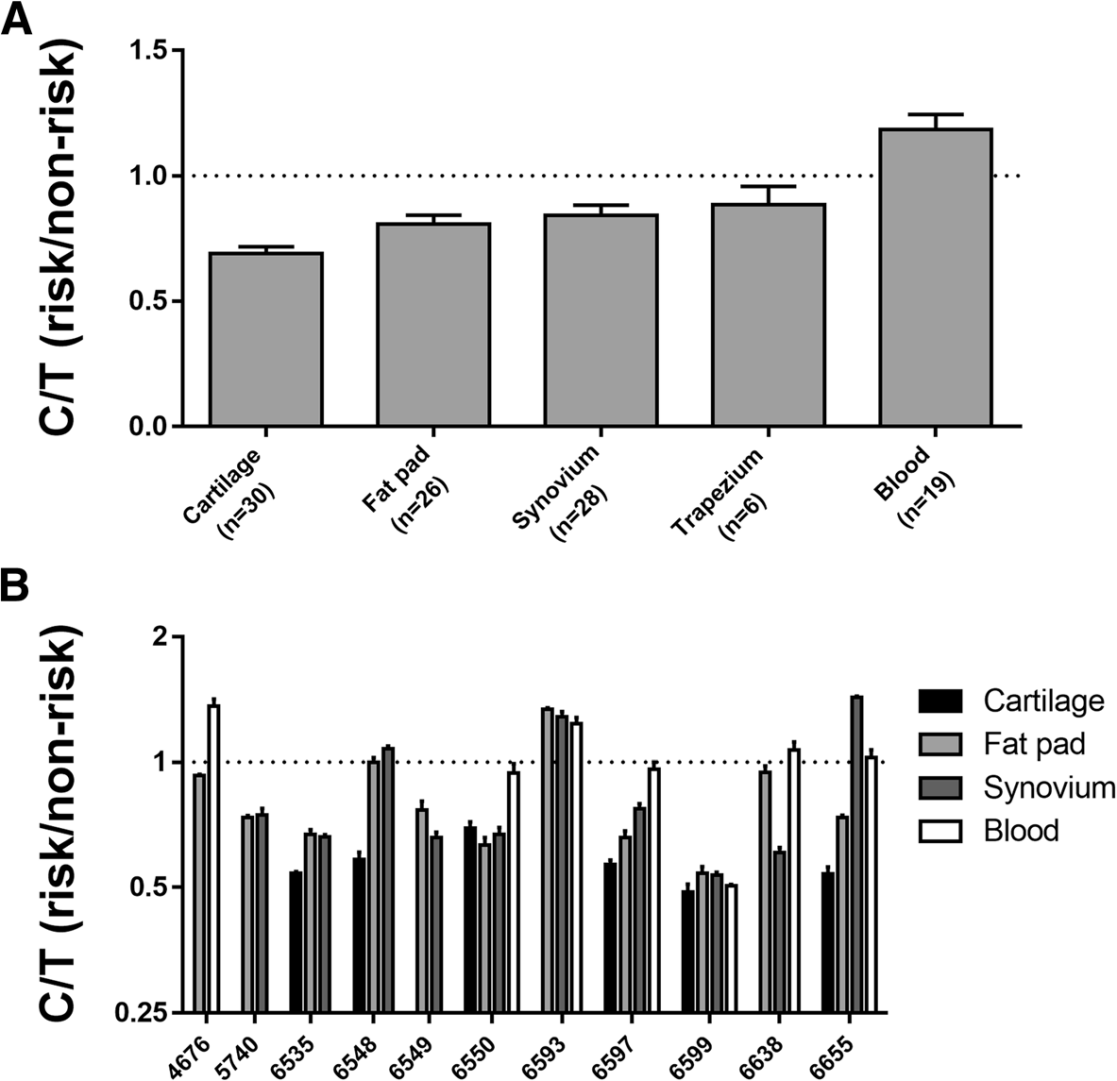
Difference in AEI between tissues and within the tissues of the same patient. **a** The mean AEI value (± SEM) was plotted for each of the five tissue types. **b** AEI analysis of 11 patients for whom samples were available from at least two tissues. Values are the mean ± standard deviation (SD) AEI plotted for each individual in each tissue tested. Patients are designated by their anonymised ID number. In both plots, the broken horizontal line indicates a C/T ratio of 1, which is indicative of no allelic imbalance

To highlight this difference in *MGP* AEI between tissues, we plotted together the mean AEI values for the five tissue types tested (Fig. 3a). This clearly shows the variation in AEI between the tissues, with the risk allele showing reduced expression in cartilage toward increased expression in blood. To emphasise the variability of AEI in tissues from the same patient, we next plotted the data for those patients in whom we had studied two or more tissues (Fig. 3b). There were 11 such patients, and there is clear variability in the C/T ratio between the different tissues tested for most of these, with patient 6597 demonstrating particularly clearly the inter-tissue variability of the ratio.

### Knockdown of *MGP* and matrix Gla protein

**Fig. 4:**
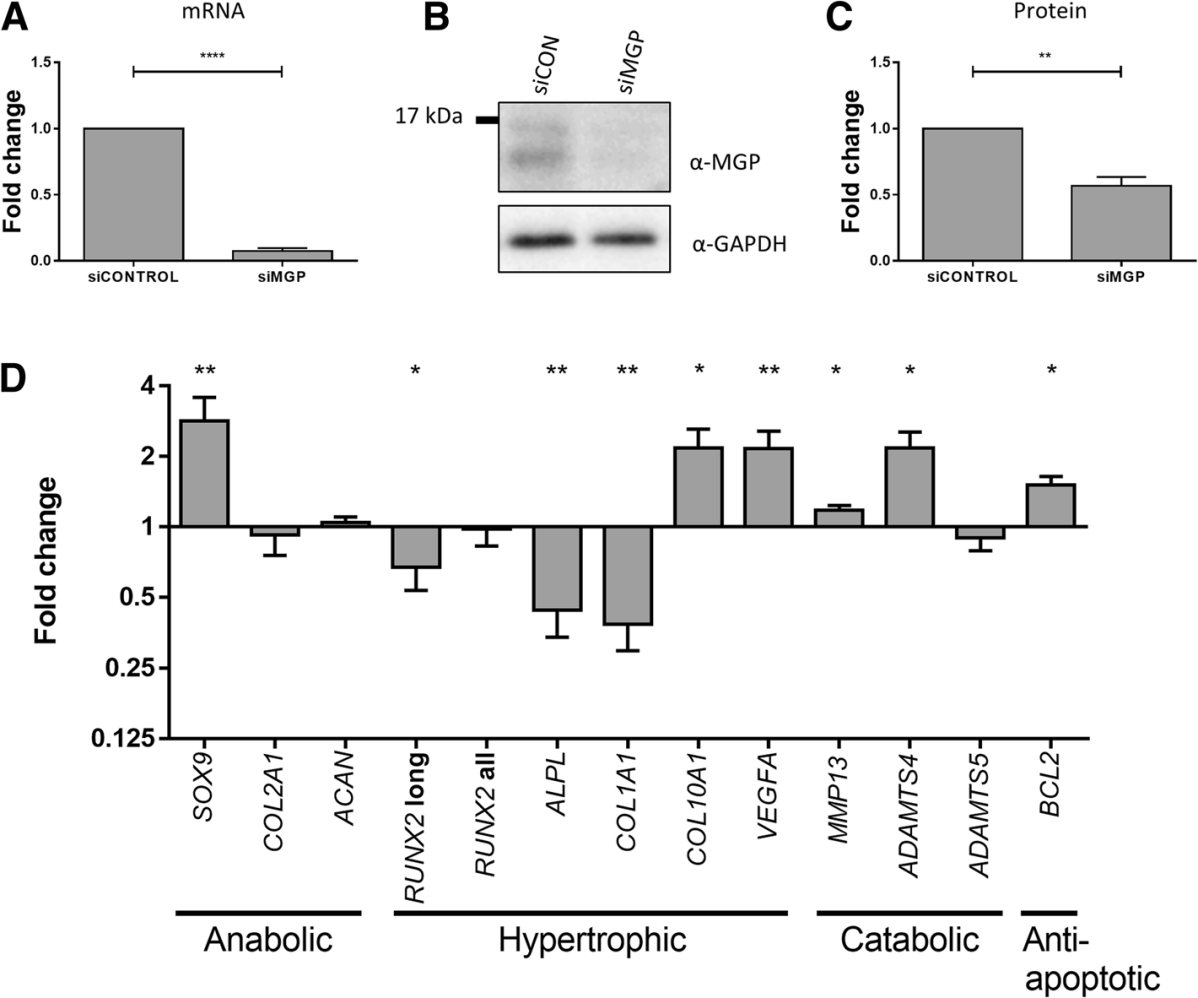
Effects of knockdown of *MGP* in primary chondrocytes from patients with OA. **a** *MGP* knockdown with siRNA targeting *MGP* (siMGP) was carried out in cultured knee chondrocytes from nine OA patients, in comparison to the effects of a nontargeting siRNA control (siCONTROL). *MGP* mRNA levels were measured by qPCR. Values represent the mean ± standard deviation (SD) fold change in expression compared to siRNA control, with the data combined for the patients. **b** Representative results from immunoblotting demonstrating depletion of matrix Gla protein. GAPDH was used as the loading control. **c** Fold change in matrix Gla protein level with the immunoblotting data combined for the patients. Values represent the mean ± SD fold change compared to siRNA control. **d** Fold change in expression of genes following *MGP* knockdown, relative to that with the siRNA control. Values represent the mean ± SD fold change in expression compared to siRNA control, with the data combined for the patients. *P ≤ 0.05; **P ≤ 0.01; ****P ≤ 0.0001

Having confirmed that the OA risk-conferring T allele of rs4764133/C allele of rs4236 correlates with decreased expression of *MGP* in joint tissues, we next modelled this effect. Chondrocytes were isolated from the knee cartilage of nine OA patients and then cultured in monolayer and subjected to *MGP* knockdown by RNAi. Compared to the effects of the scrambled siRNA control, siRNA targeting *MGP* achieved a mean knockdown at the *MGP* mRNA level of 93% (*P* < 0.0001) (Fig. 4a) with a 43% reduction at the protein level (*P* < 0.01) (Fig. 4b, c).

We then assessed the effect of this knockdown on the expression of a panel of genes that encompass cartilage homeostasis: three chondroprotective/anabolic genes (*SOX9*, *COL2A1* and *ACAN*), five genes involved in cartilage hypertrophy (*RUNX2*, *ALPL*, *COL1A1*, *COL10A1* and *VEGFA*), three catabolic genes (*MMP13*, *ADAMTS4* and *ADAMTS5*) and the anti-apoptotic gene *BCL2*. Depletion of *MGP* correlated with a significant (*P* ≤ 0.05) alteration in the expression of nine of the 12 genes tested (Fig. 4d). There was a reduction in expression of the longer isoform of *RUNX2* (encodes runt-related transcription factor 2; fold change = 0.67, *P* < 0.05), *ALPL* (encodes alkaline phosphatase; fold change = 0.44, *P* < 0.01) and *COL1A1* (encodes the α1 polypeptide chain of type I collagen; fold change = 0.38, *P* < 0.01), and a significant increase in the expression of *SOX9* (encodes transcription factor SOX-9; fold change = 2.83, *P* < 0.01), *COL10A1* (encodes the α1 polypeptide chain of type X collagen; fold change = 2.17, *P* = 0.05), *VEGFA* (encodes vascular endothelial growth factor A; fold change = 2.15, *P* < 0.01), *MMP13* (encodes collagenase 3; fold change = 1.18, *P* < 0.05), *ADAMTS4* (encodes the aggrecanase A disintegrin and metalloproteinase with thrombospondin motifs 5; fold change = 2.17, *P* < 0.05) and *BCL2* (encodes apoptosis regulator Bcl-2; fold change = 1.51, *P* < 0.05). Therefore, in this model system, depletion of *MGP* transcript and of its protein in OA knee cartilage had a significant impact on genes encoding regulators and markers of cartilage homeostasis.

## Discussion

Our initial experiments assessed the expression of *MGP* in multiple cell types from OA patients, with a focus on joint tissues. There was expression in all five of the joint tissue types tested, with the highest level being in cartilage. In a comparison between OA and non-OA cartilage (NOF samples), there was a greater expression in OA but a still abundant expression in non-OA cartilage (mean TPM values > 10,000 and > 5000, respectively). This implies that the expression of the gene is not caused by OA but that its level increases in response to OA, perhaps as an attempt to halt the disease process. In our MSC chondrogenesis models, we observed increased expression of *MGP* during cartilage formation, reiterating that expression of the gene is part of normal cartilage formation.

We next set out to replicate the *MGP* allelic expression imbalance that the earlier study had reported on [8]. Using *MGP* transcript SNP rs4236, we also showed that the OA-risk conferring T allele of rs4764133 correlated with reduced expression of the gene in patient cartilage. The degree of AEI was highly comparable between our report and the earlier study: 40.8% and 39.6% of the risk allele, respectively. In that earlier report, the authors had also observed reduced expression of the risk allele in subchondral bone. We did not investigate this tissue in our patients, but we did observe significant *MGP* AEI in fat pad and synovium tissue and in the same direction as observed in cartilage. However, the reduction in expression of the risk allele was greater in cartilage; 40.8% of the risk allele versus 44.8% in fat pad and 45.7% in synovium. Overall, this implies that the functional consequence of the OA risk mapping to this locus, namely reduced *MGP* expression, is impacting on multiple knee and hip joint tissues and that of those tissues tested; its effect is more profound in cartilage. We also investigated *MGP* expression and AEI of *MGP* in blood from OA patients. Matrix Gla protein is synthesised by vascular smooth muscle cells, where it inhibits vessel calcification [19], and by leucocytes, where it may have a role in modulating immune responses [20]. Expression of *MGP* was readily detectable in our blood samples, and there was AEI but in the opposite direction to that seen in joint tissues, with an increased expression of the OA risk allele. Our interpretation of this is that the polymorphic DNA regulatory element that is mediating *MGP* AEI is utilised by joint tissue and blood cells but that it is acted on by different *trans*-acting factors between the cell types, leading to opposite effects on *MGP* expression. We have previously shown that the direction and degree of AEI at an OA risk gene can vary between different cell types that are concurrently collected from an OA patient [21]. Our *MGP* results support this earlier observation and emphasise the probable complexity behind the regulation of the expression of this gene. A search of the GWAS Catalog (https://www.ebi.ac.uk/gwas/) did not identify any associations between *MGP* polymorphisms and ectopic calcification diseases of the vasculature. Nevertheless, the functional effect that we have observed in blood may prioritise rs4764133 and its correlating SNPs for future analyses in such diseases.

In the GWAS that reported on *MGP*, rs4764133 was associated with hand OA and, as we have done, the investigators who reported that result then used joint tissues from patients who had undergone knee and hip arthroplasty to investigate *MGP* expression [8]. This is a pragmatic decision in that these surgical procedures are common, and as such, knee and hip joint tissues are readily available for research. In our study, in addition to knee and hip samples, we also managed to collect osteochondral tissue from patients who had undergone a trapeziectomy. This involves the removal of the trapezium joint that is located at the base of the thumb and within the wrist. From a skeletal perspective, trapezium more closely matches the phenotype used in the GWAS than knees or hips, in that it is a hand joint. Furthermore, the trapezium was one of the joints that was included in the measure of hand OA in the genetic study. *MGP* was expressed in our trapezium samples, but there was no significant AEI when the six trapezium patients who we were able to study were combined. There was however a trend and in the same direction as seen for the other joint tissues, namely reduced expression of the OA risk-conferring allele.

By knocking down the gene to deplete the levels of matrix Gla protein, we finally modelled in cartilage chondrocytes the reduced expression of the *MGP* risk allele. This had a significant effect on a range of genes involved in cartilage homeostasis. We observed an increased expression of the master chondrogenic transcription factor gene *SOX9*, but this was not accompanied by an increased expression of two of its principal target genes, *COL2A1* and *ACAN*, which code for the key structural components of cartilage, type II collagen and aggrecan, respectively. Instead, we observed an increased expression of the genes *MMP13* and *ADAMTS4*, which code for enzymes that degrade these two proteins [22, 23]. We also observed an increased expression of *COL10A1* and *VEGFA*, which are markers of cartilage ossification [24, 25]. These data suggest that depletion of matrix Gla protein may have a detrimental impact on chondrocytes, whereby several markers of catabolism, hypertrophy and ossification are elevated. We hypothesise that the observed increase in SOX9 expression may be a response to counter this by boosting anabolism. There are, however, some results from the knockdown experiment that do not align with this interpretation. For example, decreased expression of *ALPL*, which is also a marker of cartilage ossification [26]. Overall however, recapitulating the AEI by reducing the expression of MGP had clear effects on chondrocyte marker genes and demonstrates that, at least in this model system, alteration in the level of matrix Gla protein has functional consequences.

In addition to our functional studies, mutations in *MGP* have been described which cause calcification of the vascular system and cartilage. Keutel syndrome arises from *MGP* mutations and manifests as vascular calcification with skeletal abnormalities, and an *MGP*-deficient mouse model displays a similar phenotype [27]. Vitamin K, and MGP functionality, was implicated in OA when plasma vitamin K concentrations were correlated with radiographic signs of OA or pathological changes to the joint [28, 29]. Furthermore, *MGP* polymorphisms were significantly associated with OA in a candidate gene study investigating vitamin K supplementation in deficient individuals [30]. Taken together, there is now a growing body of evidence to support further exploration of *MGP* and its functional role in cartilage and matrix biology, and in the ectopic mineralisation process.

## Conclusions

We have replicated and expanded on the study that correlated expression of *MGP* with the OA association signal marked by SNP rs4764133. We confirm that the OA risk-conferring allele of this SNP demonstrates reduced expression in cartilage and in other joint tissues. We also show that in peripheral blood, this effect is reversed, highlighting complexity in the regulation of expression of the gene. In our RNA-seq data, *MGP* was more highly expressed in OA versus non-OA cartilage. Combining this observation with our AEI observations, we conclude that in response to OA, chondrocytes need to express more *MGP* but that in an individual who has inherited low expressing alleles of *MGP*, their capacity to boost the gene’s expression is attenuated. As such, the disease is more likely to progress and less likely to resolve in these individuals. Our recapitulation by RNAi of the functional effect of this genetic signal had clear impacts on chondrocyte gene expression. As such, *MGP* knockdown is an appropriate model that could now be exploited to assess the capacity of interventions to overcome the genetic susceptibility at this locus.

## Additional files

Additional file 1 **Table S1.** Details of the 165 osteoarthritis patients included in this study. (XLSX 15 kb)

Additional file 2 **Table S2.** Primers used for genotyping and AEI analysis of rs4236. (DOCX 16 kb)

## Data Availability

RNA-seq data have been deposited in the NCBI Gene Expression Omnibus [accession no. GSE111358].

## Abbreviations

AEI: Allelic expression imbalance
GWAS: Genome-wide association scan
HACs: Human articular chondrocytes
LD: Linkage disequilibrium
MSC: Mesenchymal stem cells
NOF: Neck-of-femur fracture
OA: Osteoarthritis
qPCR: Quantitative polymerase chain reaction
RNAi: RNA interference
siRNA: Short interfering RNA
SNP: Single nucleotide polymorphism
TPM: Transcripts per million
