# Supplementary material for: Expression analysis of the osteoarthritis genetic susceptibility mapping to the matrix Gla protein gene MGP
Source: Arthritis Res Ther. 2019 Jun 18;21:149. doi: 10.1186/s13075-019-1934-7 (PMC6582465; doi:10.1186/s13075-019-1934-7)
Supplement: Supplementary file 2 — Table S2. Primers used for genotyping and AEI analysis of rs4236. (DOCX 16 kb) [file 13075_2019_1934_MOESM2_ESM.docx]

**Supplemental Table S1.** Primers used for genotyping and AEI analysis of rs4236. [Btn], biotin tag at the 5' end of the primer.

| **Forward primer (5'-3')** | **Reverse primer (5'-3')** | **Sequencing primer (5'-3')** |
| --- | --- | --- |
| CTACGCCATGGTTTATGGATA | Btn-CTACAGGGGGATACAAAATCAG | AGGAAGCGCCGAGGG |
